# The pharmacokinetic-pharmacodynamic modeling and cut-off values of tildipirosin against *Haemophilus parasuis*

**DOI:** 10.18632/oncotarget.23018

**Published:** 2017-12-07

**Authors:** Zhixin Lei, Qianying Liu, Bing Yang, Saeed Ahmed, Jiyue Cao, Qigai He

**Affiliations:** ^1^ State Key Laboratory of Agriculture Microbiology, College of Veterinary Medicine, Huazhong Agriculture University, Wuhan, China; ^2^ Department of Veterinary Pharmacology and Toxicology, College of Veterinary Medicine, Huazhong Agricultural University, Wuhan, China; ^3^ National Reference Laboratory of Veterinary Drug Residues and MAO Key Laboratory for Detection of Veterinary Drug Residues, Huazhong Agriculture University, Wuhan, China

**Keywords:** wild-type cutoff, pharmacokinetic/pharmacodynamic cutoff, Tildipirosin, Pulmonary epithelial lining fluid, Haemophilus parasuis

## Abstract

The goal of this study was to establish the epidemiological, pharmacodynamic cut-off values, optimal dose regimens for tildipirosin against *Haemophilus parasuis*. The minimum inhibitory concentrations (MIC) of 164 HPS isolates were determined and SH0165 whose MIC (2 μg/ml ) were selected for PD analysis. The *ex vivo* MIC in plasma of SH0165 was 0.25 μg/ml which was 8 times lower than that in TSB. The bacteriostatic, bactericidal and elimination activity (AUC_24h_/MIC) in serum were 26.35, 52.27 and 73.29 h based on the inhibitory sigmoid E_max_ modeling. The present study demonstrates that 97.9% of the wild-type (WT) isolates were covered when the epidemiological cut-off value (ECV) was set at 8 μg/ml. The parameters including AUC_24h_, AUC, T1/2, C_max_, CL_b_ and MRT in PELF were 19.56, 60.41, 2.32, 4.02, 56.6, and 2.63 times than those in plasma, respectively. Regarding the Monte Carlo simulation, the CO_PD_ was defined as 0.5 μg/ml *in vitro*, and the optimal doses to achieve bacteriostatic, bactericidal and elimination effect were 1.85, 3.67 and 5.16 mg/kg for 50% target, respectively, and 2.07, 4.17 and 5.78 mg/kg for 90% target, respectively. The results of this study offer a more optimised alternative for clinical use and demonstrated that 4.17 mg/kg of tildipirosin by intramuscular injection could have an effect on bactericidal activity against HPS. These values are of great significance for the effective treatment of HPS infections, but it also be deserved to be validated in clinical practice in the future research.

## INTRODUCTION

*Haemophilus parasuis* (HPS) is a Gram-negative bacterium belongs to of the *Pasteurella* genus. The HPS bacterium is a common inhabitant of the upper respiratory tract in pigs, and infection is characterised by arthritis, meningitis and polyserositis [1, 2]. HPS can invade the body and cause a systemic infection, and is associated with the transportation, weaning and impaired maternal immunity. According to previous reports More than 15 kinds of HPS serovars had been described. Serovars 1, 5, 10, 12, 13 and 14 have been reported to be highly pathogenic, causing the death or morbidity. Serovars 4 and 5 are the most epidemic among isolates in China [3], and this pathogen has caused a massive worldwide economic losses in the pigs industry in recent decades.

**Figure 1:**
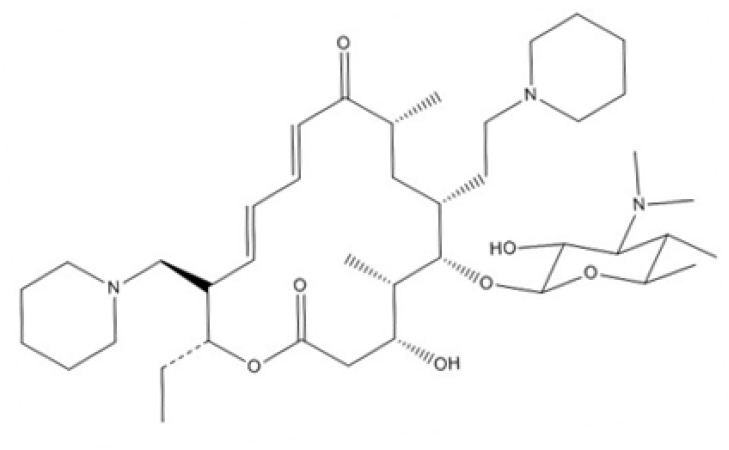
Structure of tildipirosin

Tildipirosin is a semi-synthetic tylosin analog, characterised as a novel 16-membered ring macrolide antimicrobial, depicted in Figure 1. Tildipirosin acts as a bacteriostatic against HPS and *Actinobacillus pleuropneumoniae* [4]. As a class, macrolides are characterised by extensive partitioning into tissues, where they can be found in a multi-fold higher concentration compared to the concentration in plasma [5–7]. Compared with other macrolides, tildipirosin has a long half-life and maintains a high concentration in lung tissue [8, 9].

According to the guidelines that encourage the rational use of antibiotics, the susceptibility of bacteria to antimicrobial agents should be determined prior to treatment. Presently, microbiological breakpoints (epidemiological cutoff values) and clinical breakpoints are used. Epidemiological cut-off values, in bimodal distributions of minimum inhibitory concentrations (MIC), are used to separate susceptible subpopulations from resistant subpopulations. However, microbiological breakpoints do not consider the pharmacological effects of antimicrobial agents *in vivo*. The concentration of antimicrobial agents, which can be determined at the site of infection under the recommended dosage, is an important parameter for breakpoint determination [10, 11]. The breakpoints of tildipirosin against HPS have not yet been established. Therefore it has a great importance to establish the susceptibility breakpoint of tildipirosin for both the susceptibility testing and monitoring of resistance. A multistage approach is used to develop a susceptibility breakpoints, which generally includes four steps, as follows: (1) MIC distribution is determined for a group of bacteria; (2) bacteriostatic activities are examined by time-kill experiments with selected strains; (3) available pharmacodynamic (PD) and target animal pharmacokinetic (PK) data obtained *in vitro*, in addition to efficacy data, are analysed by Monte Carlo simulation; and (4) statistical analyses of susceptibility data to determine breakpoints [12]. In previous studies, clinical data regarding antimicrobial agents have been difficult to obtain. In the absence of clinical breakpoints, epidemiological cutoff values (ECVs) can be used to help separate susceptible from resistant isolates [13, 14]. However, the susceptibility breakpoint determined from ECVs alone cannot be used to calculate the clinical breakpoint, although the PK/PD cutoff values are associated with clinical efficacy [15].

Macrolides have an extraordinary capability to accumulate in different lung tissue compartments, showing rapid and extensive distribution, in addition to persistence in pulmonary epithelial lining fluid (PELF) [7, 16]. The concentration of macrolides in PELF has been used previously to establish an intrapulmonary PK model reported by Conte [17]. However, it had been widely accepted that lung tissue was not act as the bio-phase for lung infected by pathogens [18–21]. HPS similar to *pasteurella multocida* was strictly extracellular pathogens, and the PELF was its main location for these extracellular pathogens. Although the drug concentration in PELF exceeded many times than that in plasma, it could be unable to maintain an effective local extracellular concentration in PLEF because of its extremely slow dynamic and release of drug *in vivo*. It had been reported in the previously described study by Kiem [22, 23], that high PELF drug concentration was caused by the lyses of cells (including high drug concentrations) during the bronchoalveolar lavage procedure when it was required to collect PELF. Thus, the high drug concentrations in PELF were not authentic, and it could not be the final target tissue for PK/PD analysis [20]. Furthermore, it was recommended to select PK data in plasma to study the PK/PD cutoff (CO_PD_) for macrolides.

For the evaluation of antimicrobial drugs, it is essential to optimise the dose schedule to attain the clinical cures and reduce the emergence of antimicrobial drug resistance [24]. The pharmacokinetics-pharmacodynamics (PK/PD) integration model can reveal the relationship between antibiotics and bacterium in the specific animals, and quantify the potency and efficacy of antibiotics against bacterium. Moreover, the PK/PD integration model can also prevent resistance development and provide optimal dosage strategies [25, 26]. As an effective tool for assessing the optimal dosage regimens, PK/PD analysis has been recommended in the development of new antimicrobial compounds by the Food and Drug Administration (FDA) and European Medicines Agency (EMA) [27].

The purpose of the current study was to establish the ECV and CO_PD_ of tildipirosin against HPS based on wild-type MIC distributions and PK/PD data *in vitro* and *ex vivo*, respectively, and also compare the pharmacokinetics in plasma and PELF. Moreover, the rational dosage regimen of tildipirosin against HPS was also established for veterinary clinical guidance based on PK-PD integration modeling.

## RESULTS

### Minimum inhibitory concentration distributions and wild-type cutoff values

**Figure 2:**
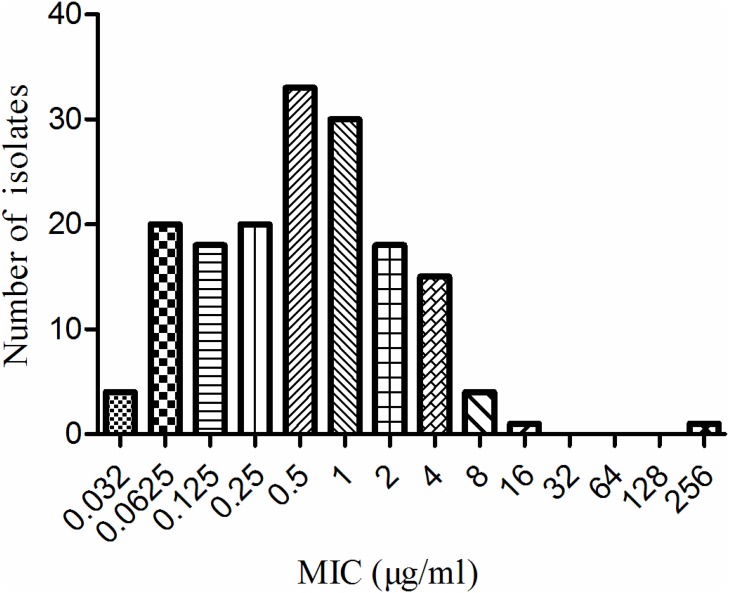
MIC distributions of tildipirosin against 164 *HPS*

The MIC distribution of tildipirosin against HPS, shown in Figure 2, was determined to be bimodal by visual inspection. The MICs of tildipirosin were distributed in the range of 0.032 to 256 μg/ml, with two peak values observed at 0.0625 and 0.5 μg/ml. The distribution ratios of each MIC (0.032, 0.0625, 0.125, 0.25, 0.5, 1, 2, 4, 8, 16 and 256 μg/ml) for the 164 isolates were as follows: “2.44% for 0.032 μg/ml, 12.20% for 0.0625 μg/ml…” and so on. The MIC_50_ and MIC_90_ were 0.25 and 2 μg/ml, respectively. All of the 164 HPS isolates were able to be identified by PCR. The MIC for chloromycetin against *Escherichia coli* (ATCC25922) was found to be 8 μg/ml, which is within and suitable for the acceptable quality control (QC) range according to the CLSI (M31-A3).

### Epidemiological cutoff values

**Figure 3:**
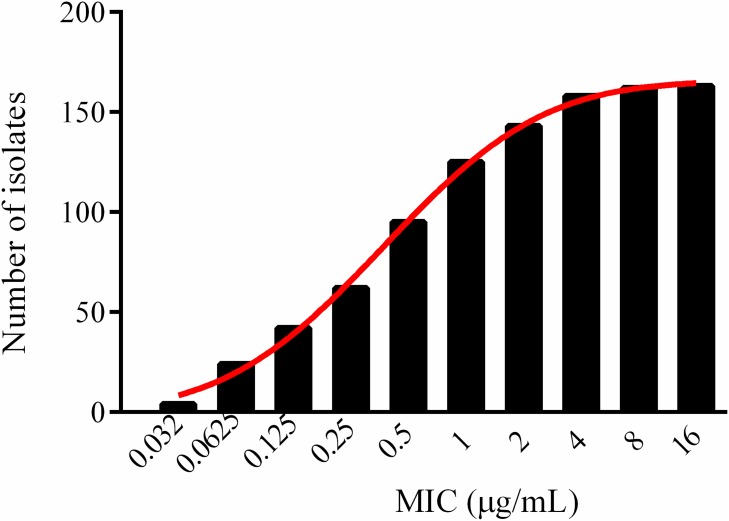
Cumulative MICs distribution of tildipirosin against HPS after non-linear regression

The cumulative counts for MIC data, with the exception of one resistant strain with an MIC of 256 μg/ml, were performed to match a suitably shaped normal distribution (normality test, *P* > 0.200) using SigmaStat and GraphPad Prism software (Figure 3). Furthermore, the strain whose MIC was 256 μg/ml had been proved as resistant one, with a resistant HAPS_RS04930 reported by Zhixin Lei [49, 50]. The optimum MIC range from 0.002 to 32 μg/ml was obtained from non-linear regression (Table 1), and the range was further corrected to 0.032 to 32 μg/ml according to the optimum distribution determined using the NORMINV function in Microsoft Excel. The probability of an MIC at 8 μg/ml was 97.9%, which encompassed 95% of the WT isolates, defined as the ECV using the NORMDIST function in Microsoft Excel (Table 2).

**Table 1:** The values of optimum non-linear least squares regression fitting

Subset fitted (μg/ml)	Number of isolates						Mean MIC(log_2_)				Standard deviation(log_2_)			
	True	Est.	Diff.	ASE	Est./ASE	95%CI^b^	Est.	ASE	Est./ASE	95%CI^a^	Est.	ASE	Est./ASE	95%CI^b^
≤0.5	95	161	66	94.780	1.7	–246.5, 569.2	–1.463	1.688	–0.9	–8.725, 5.798	2.232	0.887	2.5	0.0, 6.049
≤1	125	187	52	46.410	4.0	39.2, 334.6	–1.045	0.817	–1.3	–3.645, 1.554	2.420	0.483	5.0	0.882, 3.957
≤2	143	168	25	13.640	12.3	130.4, 206.2	–1.374	0.290	–4.7	–2.178, –0.570	2.242	0.244	9.2	1.565, 2.920
≤4	158	170	12	6.706	25.4	152.3, 186.7	–1.350	0.159	–8.5	–1.759, –0.941	2.259	0.163	13.9	1.840, 2.677
≤8	162	167	5	3.716	44.9	157.9, 176.1	–1.403	0.102	–13.8	–1.652, –1.154	2.214	0.121	18.3	1.918, 2.511
≤16^b^	163	165	2	2.489	66.3	159.6, 171.4	–1.436	0.078	–18.4	–1.622, –1.251	2.182	0.101	21.6	1.944, 2.420

Note: Est., represent the value of non-linear regression estimation; Diff., represent the value of estimation of N minus true N; ASE, represent the value of asymptotic standard error; Est./ASE, represent the value of estimation divided by asymptotic standard error; CI, represent the value of confidence intervals; a, represent the value of 95%CI of estimation; b, represent the value of the smallest difference between the estimation and the true number of isolates.

**Table 2:** The probability estimation of ECV with NORMDIST function in microsoft excel

Optimum MIC	Log_2_ Mean MIC	Mean MIC	Log_2_ SD	High cut-off (μg/ml)	Probability of a higher value
≤32	–1.436	0.370	2.182	32	99.8%
≤16	–1.436	0.370	2.182	16	99.3%
≤8^*^	–1.436	0.370	2.182	8	97.9%
≤4	–1.436	0.370	2.182	4	94.2%
≤2	–1.436	0.370	2.182	2	86.8%

Note: ^*^the epidemiologic cut-off value.

### Growth and time-killing curves and MIC of SH0165 *in vitro* and *ex vivo*

**Figure 4:**
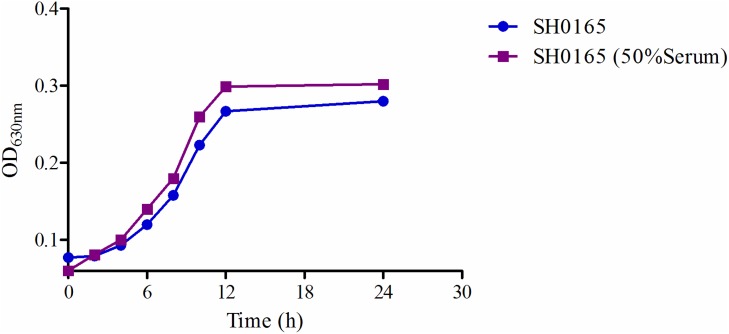
The growth-time curve of SH0165 *in vitro* and *ex vivo*

**Figure 5:**
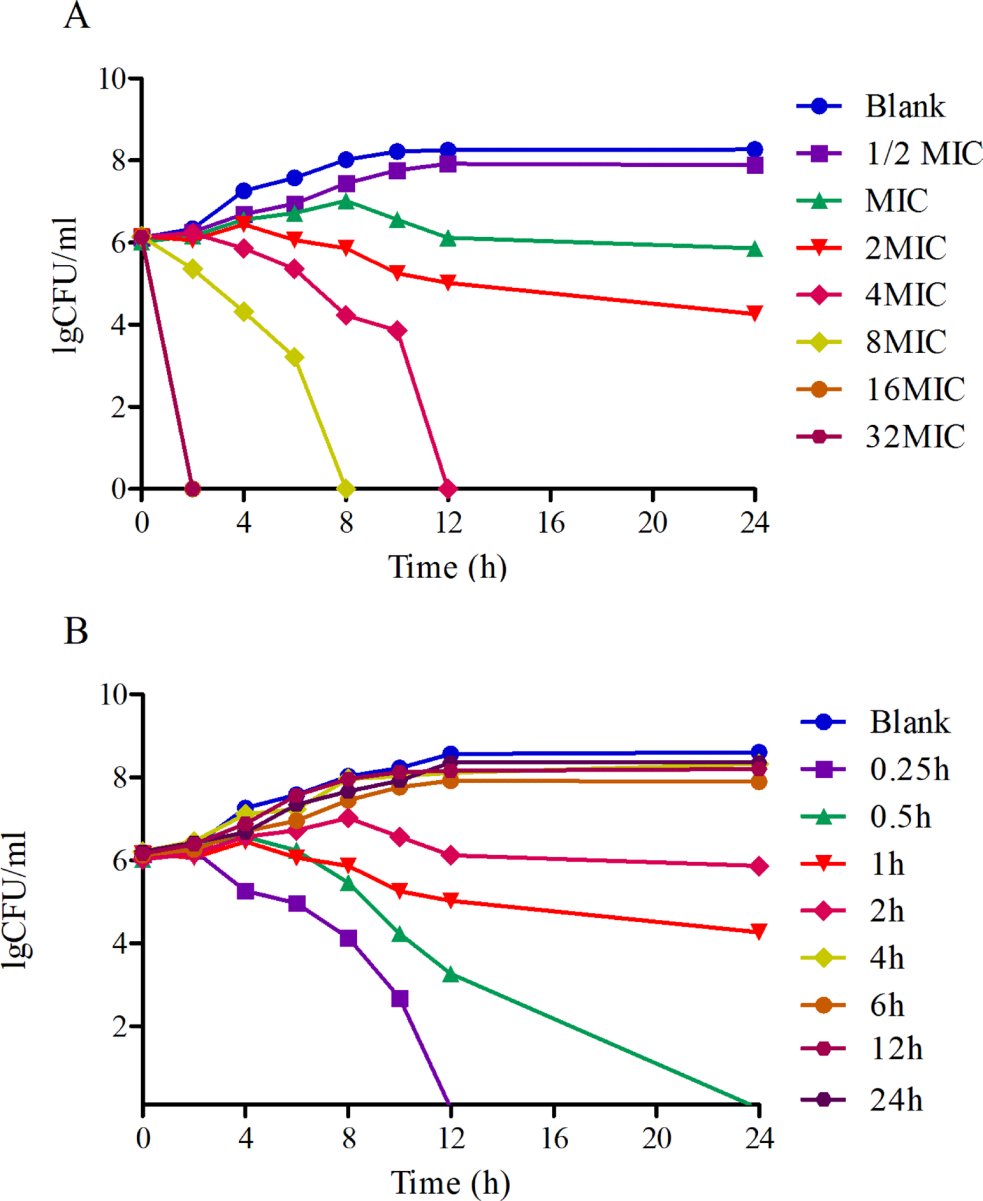
The Killing-time curves of tildipirosin against SH0165 *in vitro* (**A**) and *ex vivo* (**B**).

The logarithmic growth phase was determined to occur between 6 and 12 hours, as evaluated at OD_630nm_, shown in Figure 4. Compared with other bacteria like *E. coli*, *Streptococcus* and *Pasteurella multocida*, HPS required a longer time to reach its logarithmic growth phase. The time-killing curve of tildipirosin against SH0165 (Figure 5A) demonstrated that lower concentrations (≤ 2MIC) showed similar antimicrobial activity for HPS. The bacteriostatic efficiency gradually strengthened with increasing tildipirosin concentrations, when tildipirosin concentrations were higher than 2MIC. According to the characteristics of the killing-curve *in vitro* (Figure 5A), the activity of tildipirosin against HPS was identified as being concentration-dependent.

For *ex vivo* growth and time-kill curve, the 50% serum added into TSB was the highest limit for culturing HPS (SH0165). The serum drug concentration at 0.25 and 0.5 h (1.29 and 0.89 μg/ml) could eradicate bacterium completely (Figure 5B). This result was similar to the time-killing curve *in vitro*, and these results revealed that tildipirosin was a typically concentration-dependent drug both *in vitro* and *ex vivo*.

### Validation of the high-performance liquid chromatography method

**Figure 6:**
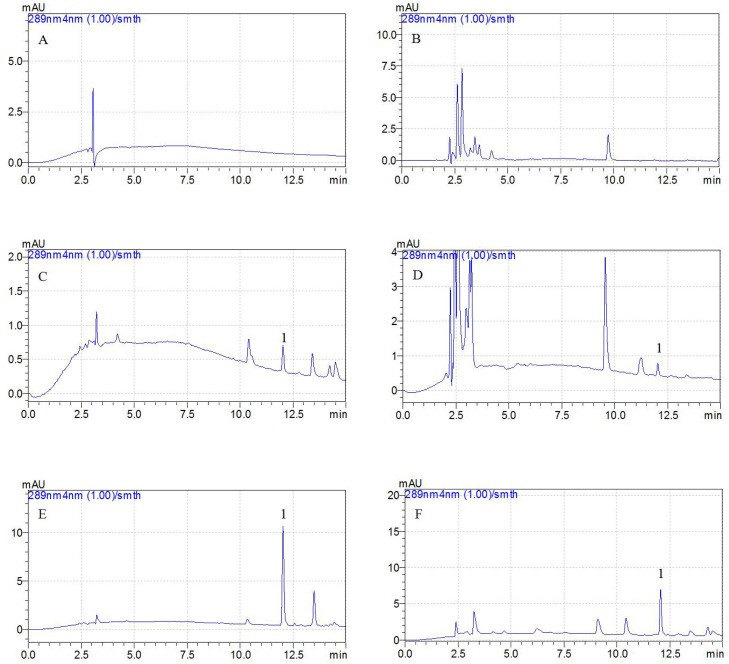
Representative HPLC chromatograms: A, blank plasma sample; B, blank BAL sample; C, plasma sample at the LLOQ of 0.01 μg/ml (5X concentration); D, BAL sample at the LLOQ of 0.05 μg/ml; E, plasma sample after i.m. administration of tildipirosin at the point of 15 min; F, BAL sample after i.m. administration of tildipirosin at the point of 1 h; 1, tildipirosin at the peak time of 12.2 min

The calibration curves showed a high degree of linearity for tildipirosin in the range of 0.05–10 μg/ml for both the plasma and bronchoaveolar lavage (BAL) samples. The correlation coefficients (*R*^2^) for tildipirosin were determined to be 0.9989 and 0.9996 in the plasma and BAL samples, respectively. The lower limit of quantification (LLOQ) value of tildipirosin was 0.05 μg/ml in the plasma and BAL samples, which appeared to be obtained with sufficient precision and accuracy. The typical chromatograms of blank samples, samples at LLOQ and samples after i.m. administration are presented in Figure 6, in which good separation of tildipirosin could be observed. The accuracy and precision were within the tolerated limits for the (QCs), ranging from 0.05 to 10 μg/ml. The inter-day variation was determined to be in the range of 0.52–1.03% and 0.25–0.68% for the BAL and plasma samples, respectively. The recovery ratios were in the range of 86 ± 1.05% to 104 ± 0.92% in BAL and 84 ± 1.02% to 102 ± 0.53% in plasma, respectively. These results suggest that the accuracy, precision, recovery and stability tests met the requirements for quantitative determination in biological samples.

### Comparison of pharmacokinetic characteristics of tildipirosin in plasma and pulmonary epithelial lining fluid samples

**Figure 7:**
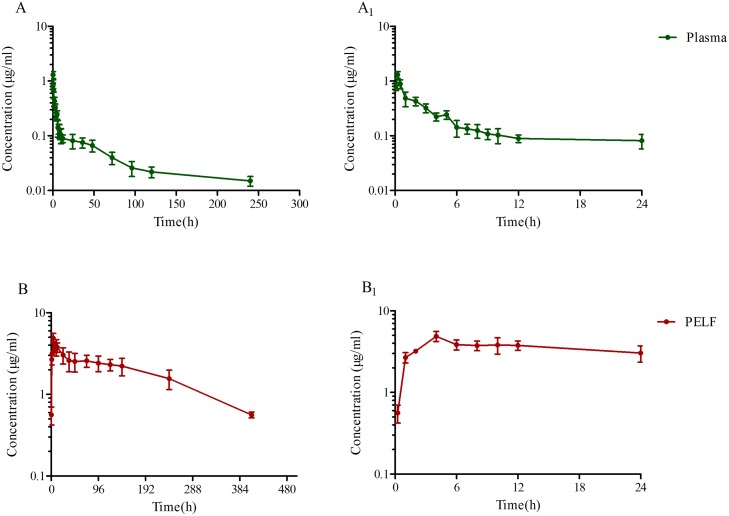
Tildipirosin concentration in porcine plasma (**A**), PELF (**B**) after i.m. administration at a dose of 4 mg/kg body weight.

The mean PK parameters in plasma and PELF after i.m. administration of tildipirosin (4 mg/kg body weight) were summarised in Table 3, including the mean and SD for tildipirosin concentrations in plasma and PELF over time. The tildipirosin concentrations were plotted on a semi-logarithmic graph, presented in Figure 7A and B. No serious adverse effects were observed in pigs after i.m. administration of tildipirosin. While plasma concentrations were eliminated below the LLOQ after 48 hours, tildipirosin concentrations in PELF reached its peak at 5.33 hours, and remained at a high concentration until 408 hours, with a low elimination rate. The ratios of urea in BAL to serum (Urea_BAL_/Urea_Serum_) were determined between five and eight times with an automatic dry-type biochemical analyser. A non-compartment model and absorbing two – compartment open model were selected to analyse the drug concentration characteristics for plasma and PELF samples. The PK parameters of tildipirosin in plasma and PELF were calculated using WinNolin software. The results for the area under the curve within 24 h (AUC_24h_), the area under the curve (AUC), the time to peak concentration (T_max_), terminal half-life (T_1/2_) of tildipirosin, peak concentration (C_max_), relative total systemic clearance (CL_b_)and the mean residence time (MRT) in the plasma and PELF were shown in Table 3.

**Table 3:** Comparison pharmacokinetic parameters in plasma and PELF

Parameters	Plasma	PELF	Ratio of PELF/Plasma
AUC_24h_ (μg^*^h/ml)	4.25 ± 0.60	83.13 ± 11.26^**^	19.56
AUC	14.16 ± 2.12	855.46 ± 76.25^**^	60.41
T_max_ (h)	-	5.33 ± 2.37	-
T_1/2_ (h)	73.39 ± 6.62	170.91 ± 18.41^**^	2.32
C_max_ (μg/ml)	1.01 ± 0.18	4.06 ± 0.65^**^	4.02
CL_b_ (L/h)	0.283 ± 0.109	0.005 ± 0.001^**^	56.6
MRT_last_ (h)	93.24± 12.11	245.19 ± 28.61^**^	2.63

Note: ^*^represent statistical significance (*p* ≤ 0.05), ^**^represent extreme significance (*p* ≤ 0.01). AUC_24h_, area under the curve within 24 h, AUC, the area under the curve, T_max_, the time to peak concentration, T_1/2_, the eliminate half-life, C_max_, the peak concentration, CL_b_, relative total systemic clearance, MRT, the mean residence time

The C_max_ of tildipirosin in plasma and PELF were 1.01 and 4.06 μg/ml, respectively, with the C_max_ for PELF determined to be four-fold higher than that in plasma. The T_1/2_ of tildipirosin in plasma (73.39 h) was much shorter than in PELF (170.91 h). However, the CL_b_ in plasma (0.283 L/h) was much higher than that in PELF (0.005 L/h), suggesting that tildipirosin was released and eliminated slowly and remained high concentration in PELF. The AUC_24h_ of PELF was 83.13 μg*h/ml, which was 19.56 folds higher than that in plasma (4.25 μg*h/ml). Significant differences in MRT, Cl_b_, C_max_, AUC, AUC_24h_ and T_1/2_ (*p* ≤ 0.01) were observed in plasma and PELF. Both pharmacokinetic parameters in PELF were higher than those in plasma apparently (Table 3).

### PK/PD integration analysis

**Figure 8:**
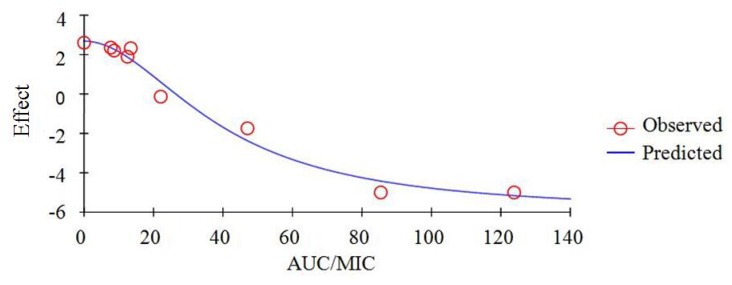
Plots of *ex vivo* AUC/MIC ratios versus the amount difference of tildipirosin against SH0165 within 24 h

As a concentration-dependent antibiotic, the selected PK/PD parameters achieved from PK data *in vivo* combined with *ex vivo* MIC were showed in Table 3. The ratios of C_max_/MIC and AUC_24h_/MIC were 4.06 and 17.12 h, respectively, based on PK/PD data in plasma (Table 3). *Ex vivo* antibacterial activity of tildipirosin against HPS (SH0165) was determined in ileum content samples collected before and at 0, 0.5, 1, 2, 4, 6, 12, 24 h after i.m. administration. The relationship between antimicrobial efficacy and the *ex vivo* PK/PD parameter of AUC_24h_/MIC ratios were simulated by using the inhibitory sigmoid E_max_ model. The model parameters of the Hill coefficient N, E_0_, E_max_ and AUC_24h_/MIC values were shown for three levels of growth inhibition in the Table 4 and Figure 8. The values of AUC_24h_/MIC ratio needed for bacteriostatic activity (E = 0), bactericidal activity (E = –3), and bacterial elimination (E = –4) were 26.35, 52.27 and 73.29 h shown in Table 4. Thus, the PK/PD targets (AUC_24h_/MIC) for CO_PD_ analysis was 52.27h when it appeared bactericidal activity (E = –3).

**Table 4:** The main *ex vivo* parameters of PK/PD modeling of tildipirosin in plasma

Parameters	Unites	Mean ± SD
AUC_24h_/MIC	h	17.12 ± 1.27
C_max_/MIC	-	4.06 ± 0.68
_Emax_	LgCFU/ml	2.48 ± 0.27
_E0_	LgCFU/ml	-5.99 ± 0.34
E_max_-E_0_	LgCFU/ml	8.47 ± 0.98
EC_50_	h	61.92 ± 6.12
N	-	1.96 ± 0.92
AUC_24h_/MIC for bacteriostatic (E = 0)	h	26.35 ± 2.32
AUC_24h_/MIC for bactericidal (E = –3)	h	52.27 ± 5.15
AUC_24h_/MIC for eradication (E = –4)	h	73.29 ± 6.45

Note: E_max_, presented the Lg change in bacterial counts of blank sample, E_0_, presented the maximal antibacterial effect, EC_50_, presented the value to achieve 50% maximal antibacterial effect, N, presented the hill coefficient.

### Estimation of doses

**Figure 9:**
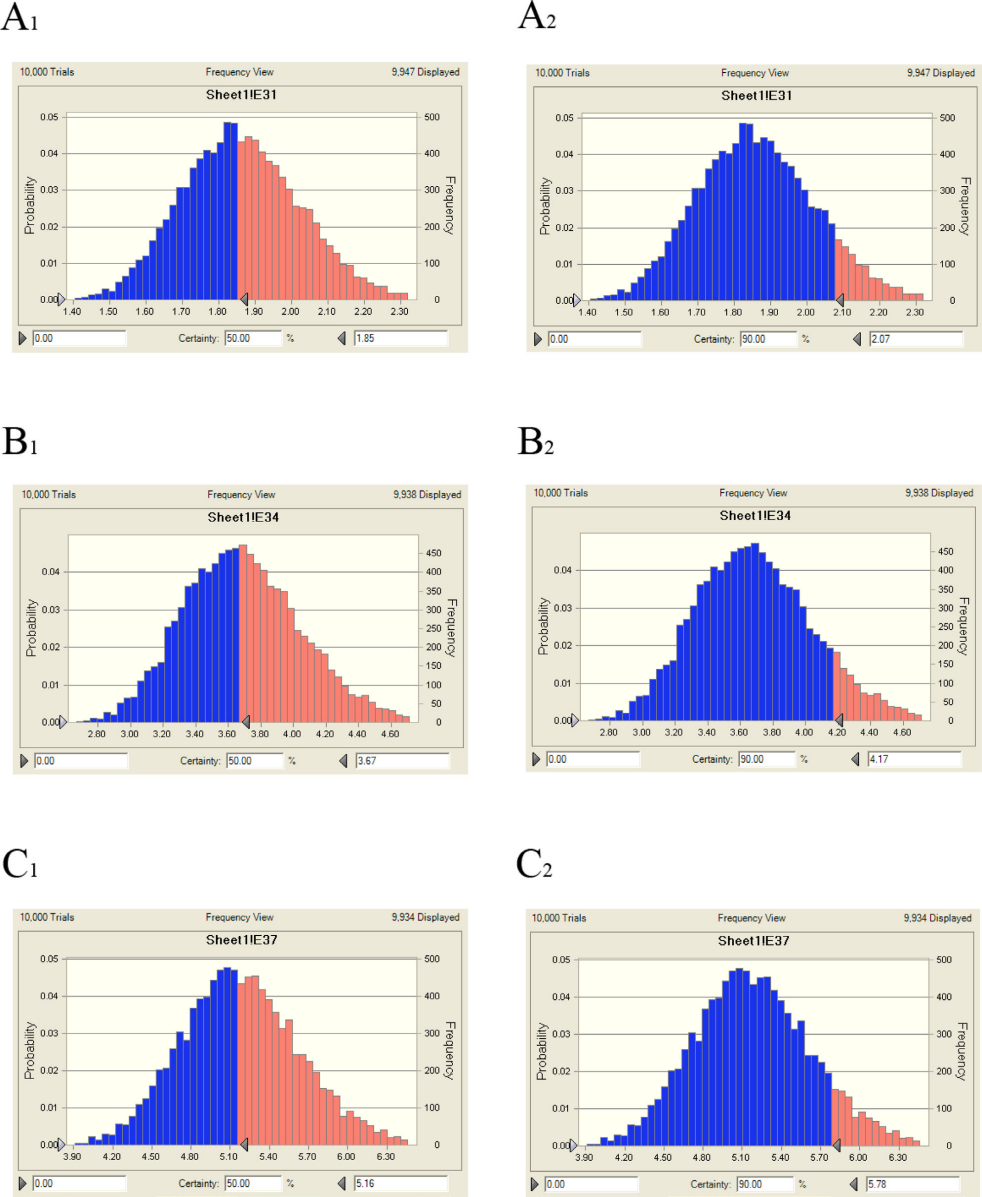
Distribution of predicted population doses (AUC_24h_/MIC) of tildipirosin against HPS (SH0165) (**A_1_**) presented the predicted population dose for bacteriostatic at 50% target, (**A_2_**) presented the predicted population dose for bacteriostatic at 90% target, (**B_1_**) presented the predicted population dose for bactericidal at 50% target, (**B_2_**) presented the predicted population dose for bactericidal at 90% target, (**C_1_**) presented the predicted population dose for elimination at 50% target, (**C_2_**) presented the predicted population dose for elimination at 90% target.

The predicted once daily doses were shown in Table 5 according to the AUC_24h_/MIC ratios and CL_b_ for these three levels of antibacterial activity calculated from the PK/PD integrating model and the distribution of *ex vivo* MIC using Monte Carlo Simulations in Oracle Crystal Ball. The distribution of predicted population dose (AUC_24h_/MIC) values of tildipirosin curing HPS for 50% and 90% targets could be observed, respectively, in Figure 9. In this study, based on the dose equations, the predicted doses for bacteriostatic, bactericidal and elimination activity of tildipirosin against HPS over 24 h were 1.85, 3.67 and 5.16 mg/kg.bw for 50% target, respectively, and 2.07, 4.17 and 5.78 mg/kg.bw for 90% target, respectively in (Table 5).

**Table 5:** The predicted daily doses of tildipirosin curing HPS

Predicted doses (mg/kg.bw)	Target ratios	
	50%	90%
Bacteriostatic (E = 0)	1.85	2.07
Bactericidal (E = –3)	3.67	4.17
Eradication (E = –4)	5.16	5.78

### Monte Carlo simulation and pharmacodynamic cut-off

Using the *ex vivo* PD and PK data determined from plasma, 10,000 Monte Carlo simulations were run using Crystal Ball software. The cumulative PTA was determined for target “52.27h” (AUC_24h_/MIC) at different WT MICs in 50% serum of pigs, as presented in Table 6. The PTA was only 0.12% at an value of 0.125 μg/ml, but reached 96.54% when the MIC value was 0.0625 μg/ml. Consequently, a PTA ≥ 90% could be obtained for isolates with MIC ≤ 0.0625 μg/ml in pig serum (50%) after i.m. administration at a dose of 4 mg/kg body weight. Based on the predicted doses of tildipirosin against HPS for bactericidal (E = –3) and elimination activity (E = –4), the PTA were calculated to be 48.23% at the MIC value of 0.25 μg/ml but 100% at the MIC value of 0.125 μg/ml, and 0.07% at the MIC value of 0.5 μg/ml but 99.79% at the MIC value of 0.25 μg/ml (Table 6). Therefore, the CO_PD_ for tildipirosin against HPS would be 0.125 and 0.25 μg/ml (*ex vivo*) after i.m. administration at a dose of 4.17 and 5.78 mg/kg body weight. However, the predicted doses required to be verified in the clinical practice, and the detail PK data also needed to obtain in the pigs. Therefore, the CO_PD_ for tildipirosin against HPS (0.0625) μg/ml at the 50% serum of pigs (*ex vivo*) was finally selected to use after i.m. administration at a dose of 4 mg/kg. According to the ratio of MIC *in vitro* to *ex vivo* (8 times), the CO_PD_ for tildipirosin against HPS was 0.5 μg/ml determined *in vitro* (TSB).

**Table 6:** The AUC_24_/MIC values calculated with Monte Carlo simulation at the predicted doses (E = –3, and E = –4)

Doses (mg/kg)	Effect	MIC (μg/ml)					
		0.03125	0.0625	0.125	0.25	0.5	1
4	-	100%	96.54%^*^	0.12%	0%	0%	0%
4.17	Bactericidal (E = –3)	100%	100%	100%^*^	48.23%	0%	0%
5.78	Elimination (E = –4)	100%	100%	100%	99.79%^*^	0.07%	0%

Note: ^*^represent the value of CO_PD_ breakpoint

## DISCUSSION

In current study, the MICs of 164 HPS isolates were tested according to the recommendations of CLSI. The WT distribution of MICs of tildipirosin against HPS ranged from 0.032 to 16 μg/ml, however, one isolate had a MIC of 256 μg/ml, which had a wider scope than previously measured [9, 51]. The MIC_90_ (2 μg/ml) measured in this study was higher than that of determined by Zuprevo for certain strains (1 μg/ml), as detailed in the CVMP assessment report [4, 52, 51]. Wild-type isolates should not have acquired any resistance mechanisms, and therefore, this isolate should be removed [53, 54]. The high MIC value (256 μg/ml) may be explained by cross-resistance of macrolides, exposure to precursors of tildipirosin (tylosin and tilmicosin) or misuse of tildipirosin. The resistance mechanism of HPS to tildipirosin or other macrolides may be associated with the pathways of amino acid ATP-binding cassette (ABC) transporter system (HAPS_2069, HAPS_RS03630) and the metabolite transporter superfamily (HAPS_2067 and HAPS_2068, HAPS_RS08950) [49, 50, 55, 56], which will be a focus of future studies. Hence, all HPS isolates (163) except one (256 μg/ml) tested in this study could be considered as a WT isolates for ECV. The measured MIC in TSB or serum and the ratio of serum to TSB were compared. The MIC in serum (50%) and TSB of SH0165 were 0.25 and 2 μg/ml, and the ratio of serum to TSB (1:2) was the limit condition for HPS which was a fastidious bacteria. The MIC in TSB was 8 times higher than that in serum. This revealed a very strong serum effect on the potency of tildipirosin, as also had been found in the previously reported by Toutain and Godinho [20, 57, 58]. It was detected broth : serum MIC ratio (up to 16) in the presence of 40% bovine serum reported by Godinho [57], and a lower ratio (50) but in the 100% serum reported by Toutain [19, 20]. Thus, the selection for MIC measure in TSB or serum was important, and the serum effect should be also considered in science study.

The bacteriostatic characteristic of tildipirosin against HPS revealed a concentration dependence, which is similar to that of reported on tilmicosin, according to data obtained from a time-kill curve obtained *in vitro*, which is different from most other macrolides that can exhibit time dependence [3, 59]. Moreover, The MIC values and growth curves of SH0165 in TSB and serum showed no significant difference (Figure 4), and the time-killing curves *in vitro* and *ex vivo* showed that tildipirosin was bactericidal against HPS, with a concentration-dependent type (Figure 5). The 4MIC concentration of tildipirosin could completely eliminate HPS after 12 h. All of these results demonstrated that tildipirosin have a strong antibacterial activity against HPS *in vitro* and in the serum. The parameter (T_max_ > MIC) is used to assess PK/PD for time-dependent antibacterial agents, but parameters AUC_24_/MIC and C_max_/MIC are used for concentration-dependent antibacterial agents. As a concentration-dependent antibacterial, this has been used as a clinical medication in pigs; AUC_24h_/MIC and C_max_/MIC are used to assess the relationship of PK/PD for tildipirosin in pigs.

The combination of HPLC with a tandem mass spectrometry has been previously used for the detection of tildipirosin. However, methods of extraction for tildipirosin from plasma and bronchial fluid with automated solid-phase extraction coupled are complex, inconvenience, inefficient and uneconomical [9, 51]. The current study reports a simplified HPLC method and extraction procedure. In this method, not many chemicals (acetonitrile, ammonium formate, methanoic acid, ethereal and dipotassium hydrogen phosphate solution) are required for the detection and extraction procedures. When developing the HPLC method for tildipirosin, we tested a different mobile phases, and found an efficient and easily accessible method with a simple composition 0.3% formic acid and acetonitrile) for HPLC analysis, with 200 μl dipotassium hydrogen phosphate solution (0.1 M) and 5 ml diethyl ether used for efficient extraction.

Similar to other macrolides (tulathromycin and tilmicosin), the concentration of tildipirosin in plasma was far below the MIC_90_ and ECV of WT isolates [60, 61]. As pathogens that target the respiratory system, HPS can attach to the bronchial epithelial cells, maintaining a high concentration in the extracellular fluid. Tildipirosin was found to be rapidly absorbed and extensively distributed to the site of infection. In general, macrolides are characterised by their rapid and extensive distribution, as well as their persistence in the PELF [7, 51]. In bronchial fluids (BF) obtained at post-mortem, the numbers of time points for the collected BF were equal to the numbers of animals required. [9] regarding to the previous reports it had been suggested that macrolides and ketolides accumulate in the inflammatory cells located in a region where the target pathogen causes infection. It is best to assess the clinical efficacy of macrolides at the site of infection [7]. According to previous studies, intrapulmonary pharmacokinetic examination had been performed by collecting the BAL from larger mammals using a sterilised catheter [7, 38, 43, 62]. However, the target tissue should be plasma but not PELF for the calculation of CO_PD_ for macrolides. Lung tissue was not the bio-phase for lung infected pathogens, and it had been accepted widely [21, 53, 54, 63]. HPS similar to *pasteurella multocida* was strictly extracellular pathogens, and the PELF was its main location for these extracellular pathogens. Although the drug concentration in PELF exceeded many times than that in plasma, it could be unable to maintain an effective local extracellular concentration in PLEF because of its extremely slow dynamic and release of drug *in vivo*. Moreover, the clinical relevance of PELF (the total tildipirosin concentrations) was only well understood by those working in a marketing department of a drug company but not by physio-pathologists [22]. At the same time, the high PELF drug concentration was caused by the lyses of cells (including high drug concentrations) during the broncho-alveolar lavage procedure when it was required to collect PELF. In this study, The C_max_ of tildipirosin in plasma and PELF were 1.01 and 4.06 μg/ml, respectively, with the C_max_ for PELF determined to be four-fold higher than that in plasma. The T_1/2_ of tildipirosin in plasma (73.39 h) was much shorter than in PELF (170.91 h). The CL_b_ in plasma (0.283 L/h) was much higher than that in PELF (0.005 L/h), suggesting that tildipirosin was released and eliminated slowly and remained high concentration in PELF. These result revealed that the drug concentration in PELF released very slowly and had a feebly dynamic. And it could be unable to reflect a real and effective local extracellular concentration, as had been previously reported by others. Therefore, it was recommended to select PK data in plasma to study the PK/PD cutoff (CO_PD_) for macrolides.

The mean plasma tildipirosin concentrations were above the MIC_90_ (0.25 μg/ml) in serum and CO_PD_ (0.0625 μg/ml in serum, 0.5 μg/ml in TSB) for HPS over a period of 4 h, predicted to remain high for a long period of time. The C_max_ of the mean plasma concentration (1.01 μg/ml) in this study was similar to that (0.90 μg/ml) in previously reported by Rose [9]. A lower mean PELF concentration (4.912 μg/ml) than those in BF (6.47 μg/ml) was measured in this study, which were comparatively higher than values previously reported in lung tissue (4.253 μg/ml) [9]. In addition, T_1/2_ (170.91 h) in this study was higher than that (106 h) reported by Rose. These differences may be explained by the abrasion of epithelial cells, as tildipirosin in lung tissue is passed into the BF during BF collection at postmortem with mechanical separation, leading to substantially higher concentrations and overestimation of the BF concentrations and longer elimination half-life [9, 41]. The higher elimination half-life was also caused by the lyses of cells (including high drug concentrations) during the broncho-alveolar lavage procedure. The tildipirosin concentrations in the current study were determined from the PELF obtained from the live animals with no cell injury, and therefore, concentrations could be lower than that previously reported in the BF. However, our results provide a more precise and representative drug concentration response in the PELF.

As a kind of concentration-dependent action for tildipirosin, the parameters AUC_24h_/MIC was regarded as a threshold for the successful therapeutic outcome of a few marcolides [27, 49, 64]. However, these thresholds may be different for different marcolides. There were differences in the immune status of target animals and pathogens. The published AUC_24h_/MIC was 30 h for bactericidal action in PK/PD study of tilmicosin against HPS [3]. Therefore, it was of great importance to study the PK/PD indices of tildipirosin individually. In this study, the PD data were obtained from a serum to predict the dosage regimens since it was more clinically relevant than those from broth. The ratios of *ex vivo* AUC_24h_/MIC and C_max_/MIC were 17.12 h and 4.06, respectively (Table 4). The inhibitory sigmoidal Emax model was used for PK/PD integration model and dosage prediction, and it showed a favourable correlation (0.992) between the observed and predicted antibacterial efficacy of tildipirosin against HPS (Figure 8). The *ex vivo* AUC_24h_/MIC ratios of tildipirosin requiring bactericidal action and eradication of the SH0165 were 52.27 and 73.29 h, which was higher than *in vivo* AUC_24h_/MIC (17.12 h) achieved after intramuscular injection administration (4 mg/kg). These result suggest that the recommended dosage of 4 mg/kg could not guarantee clinical efficacy against infections associated with HPS with an MIC90 of 2 μg/ml. Based on the Monte Carlo simulations, the predicted daily dose for 50 and 90% targets to achieve bactericidal effect were 3.67 and 4.17 mg/kg, respectively. The Monte Carlo simulation to predict the dosage for a clinical use had the advantage of taking into account the PK/PD parameters based on bacteriological outcome, and it could set target percentage such as 90 and 50% for simulation models for all data in relation to incidence in pigs [49, 65, 66]. However, due to the animals’ immune system also being an important factor contributing to bacterial eradication, the bacterial endpoint *in vivo* conditions may differ from the predicted dosages *ex vivo* [67].

## CONCLUSIONS

In order to summarize, tildipirosin demonstrated the fast absorption, rapid and extensive distribution in PELF, where respiratory tract pathogens are known to multiply and cause damage. The PK in PELF and plasma were compared in this study. According to the PK/PD evaluation *in vivo* and *ex vivo*, an observed CO_PD_ were calculated as 0.0625 μg/ml in serum and 0.5 μg/ml in TSB, and ECV was 8 μg/ml, respectively. The CO_PD_ suggested for breakpoint could be used to provide a better guidance and a greater clinical significance than ECV in the absence of clinical cut-off value. The combination of CO_PD_ with the PK/PD data obtained *in vivo* and *ex vivo* which o provide more significant results. According to the PK/PD parameters *ex vivo*, the single doses required to reach bacteriostatic, bactericidal, and eradication activity for 90% target were 2.07, 4.17 and 5.78 mg/kg, respectively. These result offers an alternative optimal dosage regimen (4.17 mg/kg for bactericidal and 5.78 mg/kg for eradication) and avoided the emergence of resistance for clinical veterinary use. However, further research is essential to achieve a more comprehensive insight into the PK/PD relationships of tildipirosin and these values are of great significance for the effective treatment of HPS infections, but it could also deserve to be validated in a clinical practice for evaluating the treatment effect of infected pigs in the future research.

## MATERIALS AND METHODS

### Bacterial isolates

A total of 164 HPS isolates were collected from Huazhong Agricultural University, specifically the National Reference Laboratory of Veterinary Drug Residues and State Key Laboratory of Agricultural Microbiology. These isolates had been isolated from pigs lung tissue samples obtained between 2014 and 2016 from 10 Chinese provinces, including Hubei, Anhui, Shandong and Henan, among others. The species of isolates was identified by polymerase chain reaction (PCR) with 16sRNA of *HPS*. Prior to testing the MIC, each isolate was subcultured at least three times in tryptic soy broth (TSB) and tryptic soy agar (TSA; Qingdao Hai Bo Biological Technology Co., Ltd., Shangdong, China) containing 5% newborn calf serum (Zhejiang Tianhang Biotechnology Co., Ltd., Zhejiang, China) and 10 μg/ml nicotinamide adenine dinucleotide (NAD; Qingdao Hope Bio-Technology Co., Ltd.). Tildipirosin was donated from Hubei Huisheng Biological Technology Company (Hubei, China).

### Animals

Fourteen healthy crossbred (Doroc × Large White × Landrace) pigs, about 25–30 kg body weight, were purchased from the pig breeding farm of Huazhong Agricultural University. Two out of fourteen pigs were used in a preliminary experiment to establish the PK and high-performance liquid chromatography (HPLC) methods. The other pigs were used for the formal PK studies. Pigs were allowed to acclimatise for 7 days prior to the experiment. All animal studies were conducted in compliance with the guidelines for the Care and Use of Laboratory Animals of Hubei Provincial Laboratory Animal Public Service Center (permit number SYXK 2013-0044), and the protocol was approved by the Ethics Committee of Huazhong Agricultural University.

### Antimicrobial susceptibility testing

Susceptibility determination of tildipirosin against HPS was performed using the agar dilution method in accordance with the CLSI recommendations in a previously described report [3]. Strains of HPS (2–4 μl, about 10^8^ CFU/ml) were inoculated onto TSA agar plates containing newborn calf serum and nicotinamide adenine dinucleotide, with two-fold serial dilutions of tildipirosin (0.0625–32 μg/ml). The resistance of strains over 32 μg/ml were screened to expand the range of two-fold dilutions of tildipirosin. Plates of strains were incubated in the presence of CO_2_ for 48 hours at 37°C. MICs were determined as the lowest drug concentrations that caused complete growth inhibition (100%). *Escherichia coli* (ATCC 25922) was used as the quality control (QC) strain to verify the results of the susceptibility testing.

### Determination of wild-type or epidemiological cutoff values

The wild-type (WT) cutoff (CO_WT_) value was defined for microorganisms that had not acquired resistance mechanisms against the target drug. The ECV was the population separate to drug-resistant isolates that had acquired or mutated resistant isolates [13, 28, 29]. The ECV were defined as the highest MIC for WT comprised at least 95% of each MIC distribution, according to the CLSI guidelines described in previous reports by Turnidge and Espinel-ingroff [28, 30–33]. The ECV was based on fit to a normal distribution at the lower end of the MIC range for obtaining reasonable MICs distribution, which was determined using Sigma-Stat software (version 3.5) (Systat Software Inc). The mean and standard deviation of the normal distribution was calculated for optimum non-linear least squares regression fitting of MICs, performed using GraphPad Prism (version 5.01) (GraphPad Software Inc, USA). The NORMINV and NORDIST functions in Microsoft Excel were performed to set the WT distribution cutoffs which were used to determine the MIC that captured at least 95% of that distribution [13, 34–36].

### *In vitro* and *ex vivo* growth, time-kill curves and MIC of tildipirosin against SH0165

The SH0165 isolate was chosen to determine the growth curve, using the method of plates count in 10^–1^ to 10^–5^ dilution ratio, in the range of 30–300 bacteria. According to the MIC of tildipirosin against SH0165 in the WT population (2 μg/ml), TSA plates were prepared with different tildipirosin concentrations ranging from 1/4 to 32 MIC (2 μg/ml) described in the previous report by Pengzhang. From the bacteria-containing fluid, 100 μl was diluted with normal sterile saline (10^–1^ to 10^–5^ dilution ratio), then aliquots of the last four diluted samples were dropped onto the TSA plates at 0, 2, 4, 6, 8, 10, 12 and 24 hours of culture, which was incubated in an atmosphere containing CO_2_ for 48 hours at 37°C.

TSB including 50% serum obtained from pigs was prepared as culture medium for *ex vivo* growth, time-kill curve and MIC. The methods of determination were similar to those *in vitro* described above using TSB (50% serum) instead of TSB. The bacteria (10^6^ CFU/ml) were co-incubated with ileum content samples obtained from pigs at different point (0, 0.25, 1, 2, 4, 6, 12 and 24 h) after treated with 4 mg/kg tildipirosin by i.m administration. The *ex vivo* time-killing curve *in vitro* was fitted to a PD model with the hypothesis of a decrease in tildipirosin concentration based on incubation time with inhibitory sigmoid E_max_ model.

### High-performance liquid chromatography to determine tildipirosin concentration

Quantitative analyses of tildipirosin by HPLC were performed for the first time in BAL and plasma. A C18 reverse-phase column (250 × 4.6 mm, i.d., 5 μm, Agilent, USA) was used for HPLC, which was performed with a 289 nm detection wavelength at 30°C. The mobile phase consisted of 0.3% formic acid (phase A) and acetonitrile (phase B). Plasma (0.5 ml) and BAL (0.5 ml) samples were mixed with 200 μl dipotassium hydrogen phosphate solution (0.1 mol/L), then extracted twice with 5 ml diethyl ether. The supernatants were achieved by centrifugation, then evaporated to dryness under nitrogen at 45°C, followed by resuspension in the mobile phase at concentrated 5 times volume (0.1 ml).

### PK study design and bronchoaveolar lavage collection

For the formal test, 12 pigs were randomly divided into two groups. Another two pigs were used to provide BAL and plasma for the HPLC analysis. Prior to the experiment, blank BAL and plasma samples were collected from every pig. Tildipirosin was intramuscularly administrated to the six pigs in each group at a recommended single dose of 4 mg/kg [9]. In group A, 30–50 ml samples of BAL were collected at 0, 5 and 15 minutes, then 1, 2, 4, 6, 8, 10, 12, 24, 36, 48, 144, 244, and 408 hours after administration. In group B, 3 ml blood samples were obtained at 15 and 30 minutes, then 1, 2, 3, 4, 5, 6, 7, 8, 9, 10, 12, 24 hour, 1.5, 2, 4, 6, 8 and 10 days after administration. The dose administered was the recommended dosage for PK of animals *in vivo* [9].

The BAL was performed using a previously described method using an electronic fiber-optic bronchoscope (Kangmei GU-180VET, Shanghai Kang Medical Equipment Co., Ltd. China) inserted into the right middle lung lobe, in order to determine the concentration of tildipirosin [37–40]. Atropine (0.05 mg/kg), ketamine (5 mg/kg) and propofol (3 mg/kg) were administrated intramuscularly and intravenously 30 minutes before drug administration. At each time point, 50 ml of sterile saline was infused into the lobe then aspirated into a 50 ml centrifugal tube. The plasma and BAL samples were stored on ice, then centrifuged at approximately 3000 *g* for 10 minutes before storing at –80°C until further analysis.

All animal studies were conducted in compliance with the guidelines for the Care and Use of Laboratory Animals of Hubei Provincial Laboratory Animal Public Service Center (permit number SYXK 2013-0044), and the protocol was approved by the Ethics Committee of Huazhong Agricultural University.

### Determination of tildipirosin concentrations in pulmonary epithelial lining fluid

The urea dilution method was used to estimate the volume of PELF in the BAL fluid, as previously described method [17, 41–43]. The concentration of urea in plasma and BAL were determined by the urea glutamate dehydrogenase enzymatic method, measured using an automatic dry-type biochemical analyser (DRI-CHEM NX500iVC, FUJIFILM (China) Investment Co., Ltd.) at the National Reference Laboratory of Veterinary Drug Residues (Wuhan, China). The volume of PELF (V_PELF_) in BAL fluid was derived according to equation 1, and the concentration of tildipirosin in PELF (TD_PELF_) was derived according to equation 2:

$${\text{V}}_{\text{PELF}}\;=\;{\text{V}}_{\text{BAL}}\;\times \;\left({\text{Urea}}_{\text{BAL}}/{\text{Urea}}_{\text{SERUM}}\right)$$ Eq.1

$$TD_{PELF}\;=\;TD_{BAL}\;\times \;\left(V_{BAL}/V_{PELF}\right)$$ Eq.2

V_BAL_ is the volume of recovered BAL fluid, and TD_BAL_ is the concentration of tildipirosin in the BAL fluid.

### PK/PD integration analysis

The PK data were analysed using WinNonlin software (version 5.2.1)(Certara USA). Plasma and PELF concentration data was analysed using a non-compartment model and absorbing two-compartment open model with lower Akaike's Information Criterion (AIC) values of 2.26 and 1.764, respectively.

Due to tildipirosin was concentration-dependent drug, the PK/PD indexes were AUC_24h_/MIC and C_max_/MIC. The AUC_24h_/MIC and C_max_/MIC were selected as the combined PK/PD parameters which were calculated in each dose of the time-killing curve. Inhibitory sigmoid E_max_ model was used to analyze the integration of AUC_24h_/MIC ratio *in vitro* and bacteria count change (CFU/ml) in ileum contents during 24 h incubation with WinNonlin software [44–46]. The model equation was described as follows equation 1.

$$\text{E }=\;{\text{E}}_{\text{max}}\;-\;\frac{\left({\text{E}}_{\text{max}}\;-\;{\text{E}}_{0}\right)\;\cdot \;{\text{C}}^{\text{N}}}{{\text{C}}^{\text{N}}\;+\;{\text{EC}}_{50}^{\text{N}}}$$

E, presented effect of antimicrobial agent measured as log_10_ difference of bacterial number before and after 24 h incubation *in vitro*, E_0_ and E_max_ presented the changes in log10 difference between 0 to 24 h in the control samples and containing tildipirosin samples, EC_50_, the AUC_24h_/MIC value reached 50% of the E_max_, C, presented the AUC_24h_/MIC ratio, N, presented the Hill coefficient.

### Monte Carlo analysis and determination of pharmacodynamic cutoff value

A Monte Carlo simulation (MCS) with 10,000 iterations was conducted using Crystal Ball software (version 7.2.2) (Oracl USA) based on PK parameters and calculated PK/PD targets (AUC_24h_/MIC) when it appeared bactericidal action (E = –3) [3, 47, 48]. The area under the curve at 24 hours (AUC_24h_) was assumed to be log-normally distributed for the mean values and confidence intervals (CI). The CO_PD_ was defined as the MIC at which the probability of target attainment (PTA) reached up to 90%, according to the CLSI guidelines described in previous reports by Pengzhang and Turnidge J [3, 28].

### Doses estimation

The following formula was used to estimate dosages in different magnitudes of efficiency (E = 0, no change in bacterial count, E = –1, 99.9% reduction in count, E = –3, 99.99% reduction) to deduce an optimal regimen.

$$\text{Dose}=\frac{\left(\text{AUC / MIC}\right)\;\cdot \;{\text{MIC}}_{90}\;\cdot \text{ CL}}{fu\;\cdot \text{ F}}$$

AUC/MIC indicates the targeted end-point for optimal efficacy, MIC denotes the minimum inhibitory concentration, CL shows clearance per day, *fu* indicates the free fraction of the drug in plasma, ignoring minimal binding, and F denotes the bioavailability.

The daily dose was calculated by Monte Carlo Simulations in Oracle Ball (Oracle Corporation, Redwood Shores, CA, USA).

### Statistical analysis

MIC_90_ was calculated by using SPSS software, and statistical analysis was performed with Student's *t*-test and Bonferroni revision for comparing the parameters of each group. The *p* < 0.05 and *p* ≤ 0.01 was considered to indicate statistically significant and extremely significant. ^*^p ≤ 0.05 and ^**^p ≤ 0.01.

## Abbreviations

HPS: Haemophilus parasuis
MIC: minimum inhibitory concentration
PD: pharmacodynamic
PK: pharmacokinetic
COPD: PK/PD cutoff
PELF: pulmonary epithelial lining fluid
ECVs: epidemiological cutoff values
PK/PD: pharmacokinetics-pharmacodynamics
TSB: tryptic soy broth
TSA: tryptic soy agar
HPLC: high-performance liquid chromatography
AUC24h: area under the curve at 24 hours
BAL: bronchoaveolar lavage
AUC: the area under the curve
Tmax: the time to peak concentration
T1/2: terminal half-life
Cmax: peak concentration
CLb: relative total systemic clearance
MRT: the mean residence time
PTA: the probability of target attainment
CFU: colony forming unit
